# Raptor: A fast and space-efficient pre-filter for querying very large collections of nucleotide sequences

**DOI:** 10.1016/j.isci.2021.102782

**Published:** 2021-06-24

**Authors:** Enrico Seiler, Svenja Mehringer, Mitra Darvish, Etienne Turc, Knut Reinert

**Affiliations:** 1 Department of Mathematics and Computer Science, Freie Universität Berlin, Berlin, Germany; 2 Efficient Algorithms for Omics Data, Max Planck Institute for Molecular Genetics, Berlin, Germany; 3 ENSTA, Paris, France

**Keywords:** genetics, bioinformatics, high-performance computing in bioinformatics

## Abstract

We present Raptor, a system for approximately searching many queries such as next-generation sequencing reads or transcripts in large collections of nucleotide sequences. Raptor uses winnowing minimizers to define a set of representative *k*-mers, an extension of the interleaved Bloom filters (IBFs) as a set membership data structure and probabilistic thresholding for minimizers. Our approach allows compression and partitioning of the IBF to enable the effective use of secondary memory. We test and show the performance and limitations of the new features using simulated and real datasets. Our data structure can be used to accelerate various core bioinformatics applications. We show this by re-implementing the distributed read mapping tool DREAM-Yara.

## Highlights

• Raptor is a tool to search through large collections of genomic texts
• Raptor is 12-144 times faster and uses up to 30 times less RAM than COBS or Mantis
• The Raptor index is 6-50 times faster to build
• The use of minimizers and Bloom filters makes Raptor very space-efficient

## Graphical abstract

**Figure:**
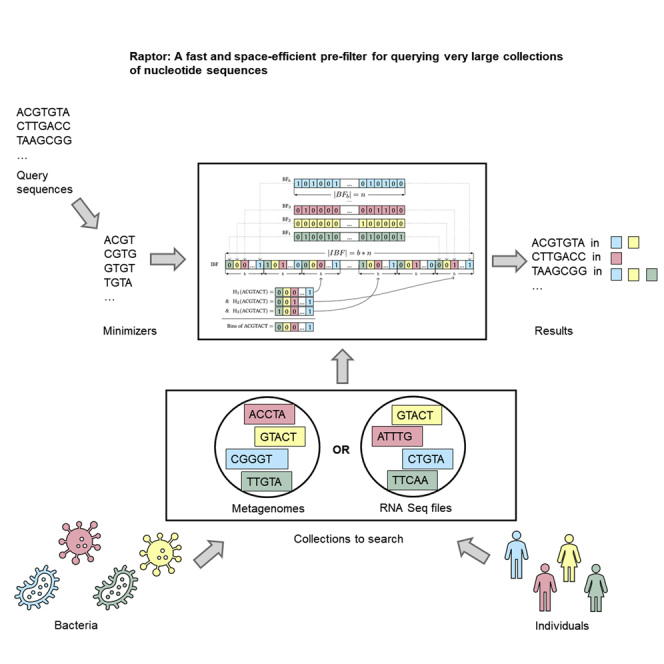

## Introduction

The recent improvements of full genome sequencing technologies, commonly subsumed under the term NGS (next-generation sequencing), have tremendously increased the sequencing throughput. Within 10 years, it rose from 21 billion base pairs (Venter et al., 2001; International Human Genome Sequencing Consortium, 2001) collected over months to about 400 billion base pairs per day (current throughput of Illumina's HiSeq 4000). The costs for producing one million base pairs could also be reduced from many thousands of dollars to a few cents. As a result of this dramatic development, the number of new data submissions, generated by various biotechnological protocols (ChIP-Seq, RNA-Seq, etc.), to genomic databases has grown dramatically and is expected to continue to increase faster than the cost and capacity of storage devices can keep up. Ongoing projects like the 100,000 Genome Project (Caulfield et al., 2019) or the American 1,000,000 Genome Project (All of Us (NIH), 2020) will easily produce data in the range of several petabases. This growth not only challenges the storage infrastructures and the processing pipelines of public databases because of the sheer data throughput but also challenges algorithm engineers to improve the efficiency of sequence analysis pipelines and develop new strategies for compression, data parallelism, and concurrent computing.

The main task in analyzing NGS data is to search sequencing reads or short sequence patterns (e.g., read mapping and variant calling) or analyzing expression profiles in large collections of sequences (i.e. a database). Searching the *entirety* of such databases mentioned above is usually only possible by searching the metadata or a set of results initially obtained from the experiment. Searching (approximately) for specific genomic sequence in all the data has not been possible in reasonable computational time. The demand for solutions can be seen by the various attempts toward enabling sequence searches on large databases (see (Marchet et al., 2019) for an overview). While the NIH SRA provides a sequence search functionality, the search is restricted to a limited number of experiments. Full-text indexing data structures, such as the FM-index, are currently unable to mine data of this scale. Word-based indices, such as those used by internet search engines, are not directly appropriate for edit-distance-based biological sequence searches (Bingmann et al., 2019). The sequence-specific solution CaBLAST (Berger and Peng, 2013) and its variants require an index of known genomes, genes or proteins, and thus cannot search for novel phenomena in raw sequencing files. This holds also true in the field of mapping-based metagenomic binning and quantitation where the relevant microbial databases grow about as fast as the sequence archives. The NCBI Refseq database of prokaryotic genomes contains about 30 GiB of sequence, still small enough to build an FM-index for the genomes, which takes about 24 hr time and about 50 GiB memory (Dadi et al., 2018). However, including the draft genomes into the analysis increases the database to 380 GiB. Building a single search structure like an FM-index for this amount of data is infeasible.

### Related work

The problem of approximately searching queries in ultra-large databases has recently been addressed by several groups, focusing on different applications, but all using methods based on the *k*-mer content of the databases. In the field of alignment-free metagenomic analysis, which focuses on *k*-mer based analysis, the size of the data also becomes slowly prohibitive. For example, Kraken (Wood and Salzberg, 2014) needs ≈147 GiB RAM for indexing 380 GBases. For analyzing RNA-Seq data, some groups aimed at searching the raw files directly for a set of transcripts ((Solomon and Kingsford, 2016) and shortly afterward (Sun et al., 2017)). They propose novel solutions to the problem of searching a transcript of interest in all relevant RNA-Seq experiments. Up until recently, these searches were only based on the sequences itself; the tool REINDEER (Marchet et al., 2020) is the first approach to also account for the sequence abundances.

As a benchmark, all three publications use a dataset of 2,652 RNA-seq sequencing runs of human blood, breast and brain tissue (a total of ≈6.5 TiB) in which they search for 214,293 known transcripts. For a *single* query their methods need in the range of 2−20 minutes, which is a tremendous improvement and a speedup of orders of magnitude compared to previous methods. Although a breakthrough, the methods presented by the groups need 4 and 0.3−2 days for processing the above set of 214,293 queries, respectively. Very recently, this time was improved by the Patro group with the tool Mantis in (Pandey et al., 2018) to 82 min. Moreover, Bradley et al. (Bradley et al., 2019) propose a Bloom filter based solution that can index about 170 TiB of (repetitive) raw sequence into an index of 1.5 TiB. However, searching, for example, 220 MiB of plasmid sequence takes 11 days using 8 cores. The same group followed up with a newer approach called COBS (Bingmann et al., 2019). Finally, the construction time of an index build on top of 170 TiB of input data was further improved by the tool RAMBO (Gupta et al., 2019) which only needs 14 hr on a cluster of 100 nodes.

Taken together, all approaches are still very demanding in terms of memory consumption and run time.

### Our contribution

In this paper, we propose a data structure, called *binning directory*, that can distribute approximate search queries based on an extension of the recently introduced Interleaved Bloom Filters (IBFs) (Dadi et al., 2018). A *binning directory* combines a so called *x*-partitioned IBF (*x*-PIBF) with winnowing minimizers and probabilistic thresholding that takes into account the varying number of minimizers in each read. We present our implementation, called *Raptor*, discuss its capabilities and limitations, and compare it with the state-of-the-art methods Mantis and COBS. Our comparison shows that Raptor is up to times faster than the competitors in answering approximate string searches with full sensitivity and a very good specificity. In addition, we only use a fraction of the memory and can further trade run time for main memory consumption.

## Results

### General method

**Figure 1:**
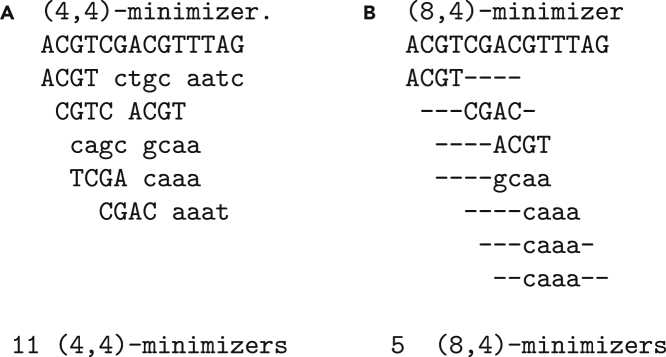
Examples of (*w,k*)-minimizers (A) and (B) show the same *k* for different window sizes, the former with a window size w=k and the latter with a larger window of 8. Note that the reverse complemented sequences, shown in lower case, have to be read from right to left. The window width is indicated by a dash and the minimizing *k*-mer is placed within the window. Subsequent windows often share the same minimizer which we illustrated by showing those as well, although they are only stored once.

**Figure 2:**
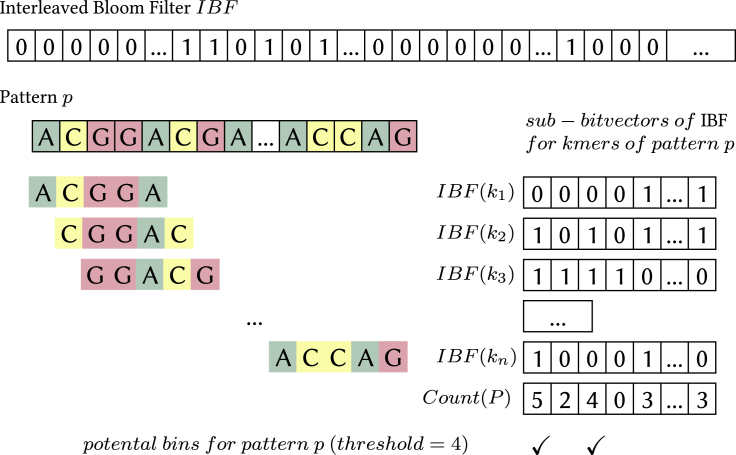
Binning directory in conjunction with the *k*-mer Lemma Bins with a counter greater than or equal to the threshold (in this case 4) need to be validated for *p*.

**Figure 3:**
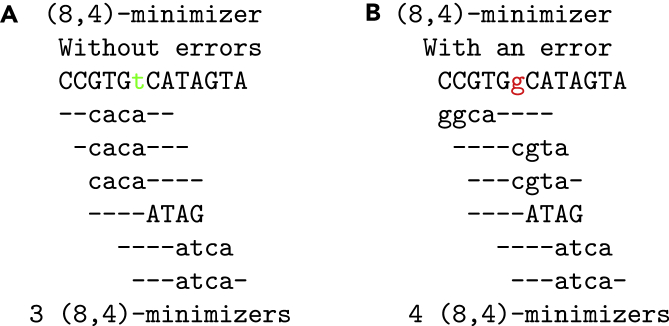
Impact of an error on the number of *k*-mers The sequences (A) and (B) represent the same sequence without and with an error at position 6 replacing a T with a G, respectively. The sequence in (A) has 3 minimizers, one of which (*caca*) is destroyed by the error position. Hence, we could assume that a sequence with one error at this position has a count of 2. However, introducing the error by replacing T with G has the effect that the first window now has a different minimizer not covering the error position (*ggca*) and hence (B) still has a minimizer count of 3. Thus, (A) and (B) would be wrongly deemed not matching with 1 error.

**Figure 4:**
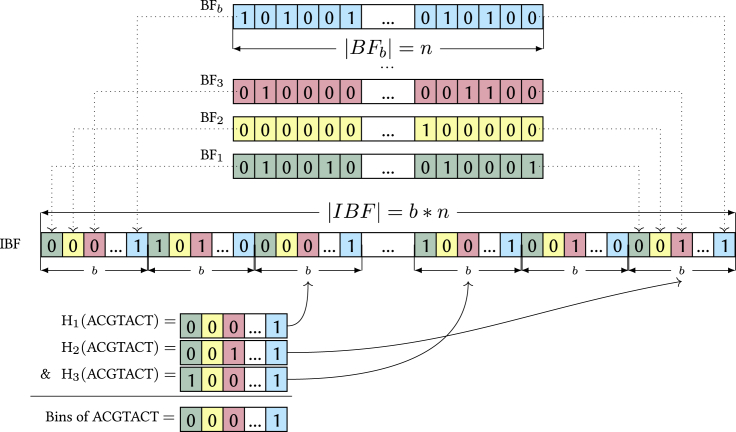
Example of an IBF Differently colored Bloom filters of length *n* for the *b* bins are shown in the top. The individual Bloom filters are interleaved to make an IBF of size b×n. In the example we retrieve 3 positions for a *k*-mer (ACGTACT) using 3 different hash functions. The corresponding sub-bitvectors are combined with a bitwise & resulting in the needed binning bitvector.

Raptor stores a *representative* transformation of the *k*-mer content of the database that is divided up into a number of bins, typically a few hundred to a few thousand (see STAR Methods section for details). The term *representative* indicates that the *k*-mer content could be transformed by a function which reduces its size and distribution (for example, using winnowing minimizers on the text and its reverse complement or using gapped *k*-mers). In this work, we use ungapped (w,k)*-minimizers* for computing representative *k*-mers (see also Chikhi et al. (2016)). A (w,k)-minimizer is essentially the lexicographically minimal *k*-mer of all *k*-mers and their reverse complements in a window of size *w*. The same transformation is applied to the *k*-mers of the query (see Figure 1 for an example and details). Raptor uses a set membership data structure, the *x*-PIBF, to retrieve *binning bitvectors* indicating whether a representative *k*-mer is in a bin or not. It then combines the binning bitvectors of all representative *k*-mers in a query into a *counting vector* and applies a thresholding step to determine the membership of a query in a bin. The process is illustrated in Figure 2, Figure 3, Figure 4.

### Evaluation

In the following, we report our computational experiments for Raptor. First, we will use an artificial dataset to discuss the limitations of binning directories, the impact of compression, the impact of the use of (w,k)-minimizers for different window sizes, and the time/space trade-off when using different partition sizes. We will also compare different binning directories with Mantis and COBS using this dataset.

Secondly, we will evaluate Raptor using a real data set used by several groups to determine the membership of transcripts in RNA-Seq files (Pandey et al., 2018) and compare Raptor with Mantis (Pandey et al., 2018) and COBS (Bingmann et al., 2019).

Lastly, we evaluated the use of Raptor in conjunction with the distributed read-mapper DREAM-Yara (Dadi et al., 2018).

All experiments were conducted on a Dell PowerEdge T640 with an Intel Xeon Gold 6248 CPU using 32 threads and 1 TiB of main memory. All file I/O for the artificial dataset was performed to and from a memory mapped file system (/dev/shm) to eliminate I/O effects, i.e., disk caching, from the measurements. Unless otherwise stated, all other I/O was performed to and from hard disk drives.

### Datasets

We created a random DNA sequence of 4 GiB size and divided it into *b* bins which would correspond to *b* different genomes in, e.g., a metagenomic data set. Using the Mason genome variator (Holtgrewe, 2010), we then generated 16 similar genomes in each bin which differ about 1% from each other on average. This could be seen as bins containing the genomes for a very homologous species. The total sequence length is hence 64 GiB, however, containing *b* groups of highly similar sequences. Finally, we uniformly sampled a total of $2^{20}$ reads of length 100 bp from the genomes and introduced 2 errors in each read to simulate a sequencing experiment. This artificial dataset is very balanced, which is the ideal case for the *x*-PIBF, since its overall size is dependent on the largest bin. We will discuss this issue in the discussion section.

On this data set, we use (19,19)-minimizers and (23,19)-minimizers in conjunction with thresholds derived by the *k*-mer Lemma or our probabilistic thresholding for determining which bin contains the query. The value k=19 was chosen to make random occurrences seldom (see STAR Methods for a detailed discussion).

In order to evaluate our method on real data, we took the dataset from (Pandey et al., 2018) which consists of 2,568 RNA-Seq experiments. Similarly to (Pandey et al., 2018), we excluded all experiments that have an average read length below 50bp because reads shorter than that are rarely relevant in practice. Furthermore, this allowed us to test the minimizer approach with a broader window size. This left us with 1,742 RNA-Seq experiments which have a size of around 6 TiB (gzipped FASTQ files). All tools were tested on this dataset using k=20, a value used in the competitors' publications which also results in an effective text ratio near 1 (see Table 8).

**Table 8:** Effective text ratios r(k)

b,k	12	13	14	15	16	17	18	19	20
64	62.84	40.52	14.19	3.96	1.57	1.13	1.03	1.01	1.00
1,024	225.98	62.19	15.92	4.08	1.58	1.13	1.03	1.01	1.00

For 64 and 1,024 bins and different values of *k* for the artificial data set. Values are rounded.

For the evaluation of DREAM-Yara, we downloaded the NCBI RefSeq for both archaea and bacteria as of February 14th 2021 and applied TaxSBP to create a taxonomic clustering of the dataset into 64 and 1024 bins. We split the RefSeq according to the clustering into 64 and 1024 gzipped FASTA files containing the sequence information of the respective clusters. Similarly to the artificial dataset experiment, we sampled $2^{20}$ reads uniformly from the bins. Within a bin, the reads were uniformly sampled from the existing sequences. We sampled reads of length 250, and introduced 2 errors in each read.

### Speed and space consumption of raptor with (w,k)-minimizers

We start by investigating the false positive (FP) count for different IBF sizes, which in turn affects the size of the individual Bloom filters in the IBF. A Bloom filter has a FP rate (FPR) depending on the ratio of stored elements to its size. For a fixed number of elements stored, it holds that the less space we allocate, the more FPs will occur. In our experiment this will lead to overcounting *k*-mers and hence lead to FP assignments of reads to bins. While one can easily compute the FPR for each individual Bloom filter (i.e. bin) of an IBF, it is harder to evaluate when a high Bloom filter FPR results in the FP assignment (FP) of a read to a bin. To evaluate this effect, we allocated IBFs of 1,2,4 and 8 GiB size and report the used RAM, construction time, search time and FP bin assignments for b=64 and b=1,024 bins for uncompressed and compressed vectors using h=2 hash functions. Also, we use (19,19)-minimizers and the traditional *k*-mer counting lemma threshold in one experiment and (23,19)-minimizers in conjunction with a new probabilistic threshold in a second experiment. The results are shown in Table 1.

**Table 1:** Run time and memory consumption of Raptor using differently sized IBF for b=64 and b=1,024

IBF		64										
		Construct		Search				Construct		Search		
		Time	RAM	Time	RAM	FP		Time	RAM	Time	RAM	FP
1 GiB	(19,19)-IBF	10:47	3,626	0.95	1,236	604,533	(23,19)-MIBF	10:14	3,577	0.92	1,238	28,438
	(19,19)-IBFc	11:28	4,456	5.54	3,382	604,533	(23,19)-MIBFc	10:50	3,570	1.80	1,986	28,438
2 GiB	(19,19)-IBF	11:37	4,693	0.91	2,260	189	(23,19)-MIBF	10:51	4,564	0.93	2,254	197
	(19,19)-IBFc	12:29	6,688	4.98	4,592	189	(23,19)-MIBFc	11:25	4,593	1.56	2,365	197
4 GiB	(19,19)-IBF	12:13	6,706	0.93	4,310	189	(23,19)-MIBF	11:05	6,703	0.92	4,310	189
	(19,19)-IBFc	13:03	10,000	4.64	5,857	189	(23,19)-MIBFc	11:52	6,924	1.66	2,750	189
8 GiB	(19,19)-IBF	12:54	10,791	0.85	8,404	189	(23,19)-MIBF	11:28	10,711	0.89	8,406	189
	(19,19)-IBFc	13:51	15,318	3.91	7,072	189	(23,19)-MIBFc	12:22	11,351	1.67	3,112	189

		1,024										
		Construct		Search				Construct		Search		
		Time	RAM	Time	RAM	FP		Time	RAM	Time	RAM	FP
1 GiB	(19,19)-IBF	4:08	6,803	3.41	1,230	9,696,884	(23,19)-MIBF	2:48	6,918	0.98	1,235	445,968
	(19,19)-IBFc	4:46	6,879	37.29	3,381	9,696,884	(23,19)-MIBFc	3:28	6,853	12.12	1,982	445,968
2 GiB	(19,19)-IBF	5:02	7,798	2.29	2,260	0	(23,19)-MIBF	3:04	7,775	0.94	2,260	141
	(19,19)-IBFc	5:48	7,807	25.39	4,592	0	(23,19)-MIBFc	3:46	7,797	8.15	2,373	141
4 GiB	(19,19)-IBF	5:40	9,964	1.92	4,308	0	(23,19)-MIBF	3:12	9,914	0.92	4,308	0
	(19,19)-IBFc	6:36	9,999	18.46	5,854	0	(23,19)-MIBFc	3:58	9,870	5.80	2,742	0
8 GiB	(19,19)-IBF	6:04	13,908	1.61	8,403	0	(23,19)-MIBF	3:21	13,999	0.94	8,404	0
	(19,19)-IBFc	7:08	15,318	12.95	7,075	0	(23,19)-MIBFc	4:13	14,044	4.59	3,112	0

On the left are the numbers for (19,19)-minimizers (IBF), on the right for (23,19)-minimizers (MIBF). Compressed versions are denoted by the suffix ’c’. Construction times are in MM:SS, search times in SS.ss. RAM represents the memory peak in MiB during the construction and search, respectively. A total of 1,048,576 reads were processed, allowing for up to 2 errors. False positives (FP) are reads originating from bin *i* assigned to a bin j≠i, neglecting the fact that the read may match with bin *j* when allowing for 2 errors.

Our experiments show that allocating only 1 GiB for an IBF using (19,19)-minimizers results in a high number of FPs for all values *b* - we would have to conduct about $6\cdot 10^{5}$ and $9.6\cdot 10^{6}$ wrong verifications for the IBF for b=64 and b=1,024, respectively. For (23,19)-minimizers, the numbers are over one order of magnitude smaller ($2.8\cdot 10^{4}$ and $4.4\cdot 10^{5}$). This is to be expected since we store a smaller set of representative *k*-mers. By doubling the size of the IBF, the number of FPs is already heavily reduced for (19,19)-minimizers. Indeed, there are no more FPs caused by the Bloom filter. Note that the 189 FP for b=64 are reads whose minimizer composition actually occurs in a different bin than its original bin by chance. Since distributing the *k*-mers to more bins reduces the chance of the same minimizer composition being present in different bins, the FP count for b=1,024 is 0. For (23,19)-minimizers, we can still see 197−189=8 FP induced by the Bloom filter for b=64 and 141 for b=1,024. This indicates that the distribution of the (23,19)-minimizers is not completely uniform or that our probabilistic threshold for the counting lemma introduced a few FP. In general, the effect of using minimizers on the FP rate is negligible. For larger sized IBF, no FP searches induced by the Bloom filter occur for both minimizer sets. The FP counts are obviously the same if we apply lossless compression to the bitvector.

Next, we look at the time and space usage for index construction. The construction time for b=64 is between 11 and 13 min and for b=1,024 between 4 and 6 min. For 1,024 bins, the wall clock time is smaller, since we can easily parallelize the construction (in chunks of 64), which is not possible for 64 bins. The time for 64 bins is larger than for 1024 bins since we insert more data in a single bin than in the case for 1024 bins. The space needed for construction is the size of the IBF and thread-local storage for the input sequences. In order to compress the IBF, both the uncompressed and compressed version must be in memory for a short amount of time, resulting in an increased memory peak.

For (23,19)-minimizers, the construction time is generally lower since we insert fewer representative *k*-mers. While for b=64, the times are comparable, the IBF can be built almost twice as fast for b=1,024 compared to (19,19)-minimizers.

Now we discuss the time and space usage for the search. For b=64, Raptor needs about 1 s to search for the $2^{20}$ reads for all IBF sizes. This holds true for both minimizer sets. Although Raptor searches fewer representative *k*-mers in case of the (23,19)-minimizers, we need to compute the minimizers of the query beforehand, which is additional work. For b=1,024 we need between 2.2 s for a 2 GiB IBF and 1.6 s for an 8 GiB IBF. The increase for larger *b* is to be expected since we need to check for all bits in the binning bitvector. This takes longer for larger binning bitvectors. For (23,19)-minimizers, we only see a slight increase and still need about 1 s. The benefit of querying fewer *k*-mers becomes pronounced and the IBF is up to twice as fast as the IBF for (19,19)-minimizers.

When searching, it is also interesting how large the memory footprint is if we use compressed bitvectors. For b=64 and (19,19)-minimizers, we see for the IBF that compression actually *increases* the memory footprint until we use ann IBF of 8 GiB. This means that the bitvectors are not sparse and that the space overhead of the compression algorithm outweighs the benefit of compressing the data.

In addition, the search time increases by a factor of about 4 for b=64 and about 8−11 for b=1,024, which makes compression here unattractive.

This changes for (23,19)-minimizers. For b=64, the search time increases from about 1 s to only 1.6 s while we can compress the bitvector from 4.3 to 2.7 GiB or from 8.4 to 3.1 GiB. For b=1,024, the compression is similar since we store the same number of *k*-mers, but the run time increases by a factor of 5−8. This is due to the need to decompress the 1,024 bit long binning bitvector which takes longer than for the 64 bit long bitvector. Still, for (23,19)-minimizers and smaller *b*, using compression offers an attractive time/space trade-off. For querying, we can observe that, in general, a sparser bitvector returns the results faster.

Finally, we investigated the impact of partitioning the IBF into x=1,2,4,8 parts. Since Raptor cannot directly evaluate the counting vector for each read after having looked at one part, we need to store the intermediate results and check if we match the threshold after having counted the *k*-mers in all parts of the partition. To do this, Raptor allocates a buffer vector of size $10^{7}$ where each position holds a vector of *b* bits that is assigned to one of the reads. After counting the occurrences of the *k*-mers of a read in one partition, we can add the result to the vector and use the vector for the next batch of reads. We report on the construction and search time, as well as on the maximum memory allocated by the resulting *x*-PIBF, for b=64 and b=1,024 bins. We use an 8 GiB IBF in these experiments. The results are shown in Table 2.

**Table 2:** Construction and search time for partitioned IBF of size 8 GiB

IBF		64										
		Construct		Search				Construct		Search		
		Time	RAM	Time	RAM	FP		Time	RAM	Time	RAM	FP
1-IBF	(19,19)-IBF	13:03	10,823	0.92	8,398	189	(23,19)-MIBF	11:23	10,773	0.89	8,405	189
	(19,19)-IBFc	13:10	15,318	4.47	7,072	189	(23,19)-MIBFc	12:04	11,351	1.41	3,112	189
2-IBF	(19,19)-IBF	16:49	6,757	1.56	4,470	189	(23,19)-MIBF	14:34	6,804	1.18	4,478	189
	(19,19)-IBFc	18:38	7,853	7.74	4,118	189	(23,19)-MIBFc	15:08	6,888	2.37	2,071	189
4-IBF	(19,19)-IBF	19:59	4,814	2.56	2,427	189	(23,19)-MIBF	19:31	4,796	1.75	2,422	189
	(19,19)-IBFc	18:43	5,055	12.86	2,339	189	(23,19)-MIBFc	19:21	4,869	4.16	1,284	189
8-IBF	(19,19)-IBF	22:35	3,930	4.27	1,405	189	(23,19)-MIBF	29:34	3,918	2.68	1,406	190
	(19,19)-IBFc	23:37	3,969	23.06	1,336	189	(23,19)-MIBFc	27:47	3,934	6.90	857	190

		1,024										
		Construct		Search				Construct		Search		
		Time	RAM	Time	RAM	FP		Time	RAM	Time	RAM	FP
1-IBF	(19,19)-IBF	6:09	13,992	1.69	8,398	0	(23,19)-MIBF	3:24	14,094	0.93	8,404	0
	(19,19)-IBFc	7:10	15,318	12.44	7,071	0	(23,19)-MIBFc	4:13	14,107	4.57	3,112	0
2-IBF	(19,19)-IBF	6:32	10,548	3.48	6,396	0	(23,19)-MIBF	4:10	10,642	1.51	6,396	0
	(19,19)-IBFc	7:36	10,542	24.94	6,037	0	(23,19)-MIBFc	5:01	10,646	8.70	4,079	0
4-IBF	(19,19)-IBF	6:48	8,490	6.29	4,348	0	(23,19)-MIBF	5:32	8,572	2.61	4,350	0
	(19,19)-IBFc	7:52	8,489	47.10	4,259	0	(23,19)-MIBFc	6:23	8,572	16.48	3,204	0
8-IBF	(19,19)-IBF	7:51	7,631	11.70	3,318	0	(23,19)-MIBF	8:25	7,512	4.62	3,318	8
	(19,19)-IBFc	8:39	7,432	92.77	3,278	0	(23,19)-MIBFc	9:19	7,512	32.07	2,772	8

The IBF is partitioned into 1 to 8 parts. On the left are the numbers for (19,19)-minimizers (IBF), on the right for (23,19)-minimizers (MIBF). Compressed versions are denoted by the suffix ’c’. Construction times are in MM:SS, search times in SS.ss. RAM represents the memory peak in MiB during the construction and search, respectively. Raptor processes a total of 1,048,576 reads, allowing for up to 2 errors. False positives (FP) are reads originating from bin *i* assigned to a bin j≠i, neglecting the fact that the read may match with bin *j* when allowing for 2 errors.

In general, we observe for all minimizers that the construction and query times grow higher the more parts Raptor uses. For (19,19)-minimizers, the build time increases from about 13 min to 23 min for b=64, while for b=1,024 it increases from about 6 to 8 min. For b=64, the search time for the IBF increases from about 1 s for a 1-PIBF to 4.3 s for an 8-PIBF. When using more parts, the run time increases, but the space needed to hold a single part in memory decreases. While we need 8 GiB memory to use a 1-PIBF, we only need 1.4 GiB if we use an 8-PIBF. When using (23,19)-minimizers, we see similar trends for b=64. Furthermore, like in the unpartitioned case, the search time is faster. Indeed, for an 8-PIBF we need only 2.68 s for the query and for an 8-PIBFc only 6.9 s while using only 857 MiB peak memory.

For b=1,024 and (19,19)-minimizers, the search times for the IBF increases from 1.69 s to 11.7 s for ann 8-PIBF. As before, compression is unattractive for this case, while it pays off for the (23,19)-minimizer version.

In general, the construction time of Raptor's index increases, the more parts we create, since we have to stream over our input *x* times and store *x* parts on the disk. However, we observe that this increase has a lower rate than the increase in the parts, as both constructing and querying an *x*-PIBF do take less than *x* times the time of a 1-PIBF. The reason for this is that we do not have to access the bitvector for *k*-mers that are not in the current part.

### Impact of probabilistic thresholding on false negatives

In this section, we show that our probabilistic thresholding is crucial in avoiding false negatives. When we use (19,19)-minimizers, the *k*-mer lemma ensures that we have no false negatives, but this is no longer true when using minimizers with w>k. This is apparent since the *number* and *distribution* of (w,k)-minimizers is sequence dependent and hence leads to a different threshold for each read. In the methods section, we describe how we derived a method to compute, for a given maximal number of errors, a threshold depending on the parameters *w*, *k* and the number of minimizers a query has.

Tools like Mantis and COBS, which use a simple percentage cutoff, would suffer in a similar increase in false negatives if they used minimizers. However, they could use our results to adapt their thresholding. In our dataset, the number of minimizers for each read ranges from 15 to 35 while our thresholds range from 4 to 13.

For example, for a query of length 100 with 20 minimizers the threshold is 6, which is lower than 0.41⋅20=8.2. Using our threshold avoids falsely filtering out the query. In general, the percentage of minimizers that need to be present ranges from 26% to 38%. This shows that applying a single threshold is not sufficient. Table 3 shows the resulting FPs and FNs of our adapted Lemma for various IBF sizes.

**Table 3:** False positives (FPs) and false negatives for differently sized (23,19)-MIBF using the adapted k-mer lemma (Lemma 2 in extra content)

IBF	64		1,024	
	FP	FN	FP	FN
1 GiB	309	796	1803	753
2 GiB	189	1803	0	1696
4 GiB	189	2270	0	2172
8 GiB	189	2422	0	2308

The resulting threshold is ≈41%. False positives are reads originating from bin *i* assigned to a bin j≠i, neglecting the fact that the read may match with bin *j* when allowing for 2 errors. False negatives are reads originating from bin *i* not assigned to bin *i*.

### Comparison with other tools

In the following, we compare Raptor with the state-of-the-art tools Mantis (Pandey et al., 2018) and COBS (Bingmann et al., 2019) using the artificial data set and a real data set of 1,742 RNA-Seq experiments also used in (Pandey et al., 2018) as described earlier. Note that the computational experiments for the real data set only use one thread for all tools, the same as it was done in the competitors' publications. The effect of parallelization was tested using the artificial data set where we used 32 threads.

We built an index over the artificial dataset (separated in 64 and 1,024 bins) with COBS and Mantis for a *k*-mer size of 19. Afterward, we queried the same reads we have already searched with Raptor using BDs. Both COBS and Mantis consider a transcript found if the amount of *k*-mers found is more or equal to a given threshold. Instead of using the default threshold of 80 percent, we determined a threshold according to the standard *k*-mer counting lemma, which was 53 percent.

Moreover, we had to adapt our input for the index construction of Mantis by adding random quality scores to our FASTA files because Mantis only accepts FASTQ files as input. But even with this adaptation, Mantis, or more precisely the helper tool Squeaker, resulted in a segmentation fault for the artificial dataset separated in 64 bins, thus we only present result for Mantis with 1,024 bins.

As can be seen in Table 4, the construction of COBS and Mantis takes at least three times longer than for Raptor. Furthermore, searching with Raptor only needs a fraction of the space (about 5−8 GiB vs. 20 GiB) COBS and Mantis need, while being as accurate. The most striking difference is the search time. For (19,19)-minimizers, Raptor needs between 0.9 s for b=64 and 1.6 s for b=1,024. This is about 144 times faster than COBS and (for b=1,024) about 30 times faster than Mantis.

**Table 4:** Comparison COBS and Mantis for the artificial dataset with 64 and 1,024 bins

IBF		Construct			Search		
		Time	RAM	Space	Time	RAM	FP
64	COBS	89:06	20,654	21	130.8	20,492	125
	Mantis	–	–	–	–	–	–
1,024	COBS	26:37	20,657	21	134.33	20,470	0
	Mantis	78:33 (+49:12)	36,048	21	46.81	21,018	0

Construction times are in MM:SS and search times in SS.ss. The construction time in brackets for Mantis is the additional time the preprocessing tool Squeaker needs. The used disk space is in GiB, the maximum RAM in MiB. All approaches are built for k=19.

Next, we evaluated the tools on the real dataset. We built an index over the dataset (that means for 1,742 bins) with COBS, Mantis, and Raptor using a binning directory for a *k*-mer size of 20 (since this value was used in (Pandey et al., 2018)). For Raptor, we created two versions, one using a binning directory with an IBF with (20,20)-minimizers and one version using a binning directory with an IBF with (40,20)-minimizers. Mantis uses a cutoff in order to sort out low-frequency *k*-mers that probably resulted from sequencing errors (Pandey et al., 2018). In order to be comparable, Raptor applied the same cutoffs for both versions of the binning directories. The results are shown in Table 5.

**Table 5:** Comparison of raptor, COBS, and Mantis

IBF	Construct		
	Time	RAM	Space
(20,20)-IBF	34	8.1	8
(40,20)-IBF	2	0.9	0.8
COBS	4,620	702.6	4,265
Mantis	135	29.7	17

Construction times are in minutes. The used disk space and the maximum RAM are given in GiB. All approaches are built for k=20.

Raptor's construction time of the binning directory is faster than Mantis and COBS. The space consumption drops to only 5% of that of Mantis when using (40,20)-minimizers. COBS's construction time and space consumption is nowhere near the other two applications, because COBS has no preprocessing step and does not use cutoffs to filter out erroneous *k*-mers. Therefore, further comparisons to COBS are omitted.

In order to compare the query times, three differently sized sets (100, 1,000, and 10,000 transcripts) were used. Each set was created by randomly picking human gene transcripts. The lengths varied between 46 bp and 101,518 bp.

The FPR was determined by comparing Raptor's results to Mantis, assuming Mantis correctly finds all experiments as it claims to be exact. A similar evaluation was applied in (Pandey et al., 2018). Also, the definition of a found transcript is based on the evaluation of (Pandey et al., 2018). Therefore, both Mantis and Raptor consider a transcript found in an experiment if 80% of its representative *k*-mers are found.

As shown in Table 6, Raptor using a BD with (40,20)-minimizers is significantly faster (12−58 times) than Mantis and uses only a fraction of main memory while still being specific with a low FP rate of about 0.017. Even when using (20,20)-minimizers, Raptor outperforms Mantis in space and time consumption.

**Table 6:** Comparison of raptor and Mantis

Transcripts		Search		
		Time	RAM	FPR
100	(20,20)-IBF	7	8	0.025
	(40,20)-IBF	1	0.8	0.015
	Mantis	12	18.7	0.0
1,000	(20,20)-IBF	10	8	0.03
	(40,20)-IBF	1	0.8	0.016
	Mantis	30	19	0.0
10,000	(20,20)-IBF	46	8	0.031
	(40,20)-IBF	4	0.8	0.017
	Mantis	232	23.3	0.0

Search times are in seconds, RAM is given in GiB. All approaches are built for k=20.

### Biological case study

To evaluate the use of Raptor in conjunction with other bioinformatics applications, we re-implemented the distributed read mapping tool DREAM-Yara (Dadi et al., 2018). These changes consisted of changing to the IBF implementation used in Raptor as well as the incorporation of minimizers. Additionally, smaller changes to address inconsistencies in command line usage and bugs where applied to both the original and new DREAM-Yara.

We will compare the original DREAM-Yara using 19-mers to the new DREAM-Yara using 19-mers and both (23,19)- and (31,19)-minimizers for different IBF sizes.

The steps involved building the FM-indices used by DREAM-Yara were not affected by the adaptations, hence the build time and memory consumption for the FM-Index in DREAM-Yara are for both new and original around 40 min and 85 GiB, respectively, for 1024 bins and 5 hr 20 min and 89 GiB, respectively, for 64 bins.

As can be seen in Table 7, the memory consumption of both versions of DREAM-Yara is virtually the same when using 19-mers and an IBF of size 16 GiB. The IBF build step uses around 17.1 GiB and 17.4 GiB of memory while the mapping requires approximately 30 and 23 GiB for 64 and 1024 bins, respectively. The new DREAM-Yara shows a performance improvement regarding run time, reducing the time needed to build the IBF from 9:17 to 7:3 and 9:14 to 7:49 for 64 and 1024 bins, respectively. The mapping time similarly decreased from 5:15 to 3:26 min and from 7:39 to 6:18 min. While the original version already had 255 (64 bins) and 220 (1024 bins) unmapped reads, the new version increased this by a handful of reads to 257 and 234, respectively.

**Table 7:** Comparison of DREAM-Yara with and without minimizers

IBF			IBF			Mapper		
	w	k	Size	Time	RAM	Time	RAM	Unmapped reads
64	–	19	16	9:17	17,129	5:15	29,913	255
	19	19	16	7:37	17,148	3:26	30,087	257
	23	19	4	4:15	4,858	3:54	17,516	257
	31	19	2	2:15	2,820	4:24	15,200	257
1024	–	19	16	9:14	17,404	7:39	23,091	220
	19	19	16	7:49	17,400	6:18	23,037	234
	23	19	4	4:12	5,082	6:59	10,865	234
	31	19	2	2:18	3,051	6:52	8,988	234

IBF sizes are in GiB, times in MM:SS, and RAM in MiB. Both the original (the *w* column contains a −) and new version of DREAM-Yara were used to build an index of the NCBI RefSeq and search for $2^{20}$ reads. All approaches use a *k* of 19, and the new DREAM-Yara additionally uses minimizers with a window length of 23 and 31, to build an IBF of a given size. Shown are the time and memory consumption for building the IBF and mapping the reads.

Applying minimizers allows using smaller IBFs, for (23,19)-minimizers, a 4 GiB IBF is sufficient, and for (31,19)-minimizers, even 2 GiB are enough. This decreases the memory consumption by around the amount of memory saved by the smaller IBF, resulting in needing around 5 and 3 GiB required to build the IBF. The memory consumption of the mapping step decreases to 17.5 GiB for (23,19)-minimizers and to 15.2 GiB for (31,19)-minimizers when using 64 bins and to 10.8 and 9 GiB when using 1024 bins. The time needed to build an IBF only varies by a few seconds between 64 and 1024, with 19-mers for the new DREAM-Yara having the biggest difference with 12 s (7:37 and 7:49 for 64 and 1024 bins, respectively), while the difference is 3 s otherwise. When using the same parameters, the new version is around 1.5 min faster to build the IBF (from 9:17 to 7:47). Using minimizers amplifies this effect, resulting in an IBF build time of 4:15 for (23,19)-minimizers and 2:15 for (31,19)-minimizers due to a smaller IBF size and less representative *k*-mers to process. The new version of the IBF is also able to answer queries faster than the version used in the original DREAM-Yara, hence the time needed for mapping decreases when switching to the newer version. Comparing for 19-mers, there is a change from 5:15 to 3:25 for 64 bins and from 7:39 to 6:18 for 1024 bins. Since the thresholds for minimizers are heuristic, this generally results in more verification being necessary, i.e. the mapper needs to map more reads. Hence, while still faster than the original version, the runtime for 64 bins increases to 3:54 and 4:24 for (23,19)- and (31,19)-minimizers, respectively. Likewise, the runtime for 1024 bins increases to 6:59 and 6:52 for (23,19)- and (31,19)-minimizers, respectively.

When using minimizers and hence a probabilistic thresholding, the number of unmapped reads increased only by 2 respectively 14 which is quite acceptable considering the memory decrease.

## Discussion

In this paper, we presented an approach to answer approximate string queries using a *representative* set of *k*-mers of the database and query. We stored a set of (w,k)-minimizers as representative *k*-mers of the database in a binning directory using a partitioned, IBF.

Binning directories could be used in various settings which we discuss below.

### Using BDs for metagenomic profiling

Tools like Kraken (Wood and Salzberg, 2014) or Centrifuge (Kim et al., 2016) perform metagenomic binning by querying the *k*-mer content of genomes using NGS reads and inferring the presence or absence of organisms in the sample using the taxonomy of a phylogenetic tree.

Hence, we could use BDs for a classification based on taxonomic levels (e.g., species, genus, …) or assembly level, and group the reference genome sequences accordingly. Using the counts for *k*-mers given by the BD, we can infer the composition of a metagenomic sample. Indeed, (Piro et al., 2020) already applied this idea as described in (Dadi et al., 2018) for this task. This resolved the problem of uneven bin sizes by applying a preprocessing step to distribute the *k*-mer content of bins more evenly.

### Using BDs for querying file content

Another application for BDs which we also used in one benchmark is to query all existing human RNA-Seq files in the SRA for the presence of transcripts. For this application, the bins would be defined by the respective file content. We would expect that the effective text size n(k) is considerably less than 5 TBases since we sample from human genes. Of course, this application scenario is not limited to RNA-Seq files.

**Table:** Example of the assignment of *q*-mers to x = 5 partitions

2-mer	AA	AC	AG	AT	CA	CC	CG	CT
Part	0	0	0	1	1	1	2	2
2-mer	GA	GC	GG	GT	TA	TC	TG	TT
Part	2	3	3	3	4	4	4	4

Given a DNA alphabet (*σ* = 4) and x = 5, we have to distribute the 16 possible *q*-mers evenly to the 5 parts. In this example we assume a uniform distribution of the *q*-mers.

### Using BDs for read mapping

In the context of read mapping, we can use the BD as follows. The database would be the reference genome(s) we want to map our reads against. Assume we have divided them into bins such that similar parts of the genomes are placed within the same bin. In the context of metagenomics analysis, this could be achieved by using a taxonomic tree (see also (Dadi et al., 2018)); alternatively, the sequences could be clustered based on similarity. The sequences in the bins could then be indexed using a compressed suffix array or other suitable indices and the BD can be used to distribute the approximate searches. The biological case study in this paper is an example of such an application.

### Possible extensions

Currently, Raptor stores all representative *k*-mers, even if some representative *k*-mers in the reference dataset are ubiquitous, i.e. they appear in all or almost all, e.g., 95%, bins. While some approaches, like Mantis, exclude certain *k*-mers from consideration, one could instead exclude them from the IBF and store them in a small lookup table. Whenever such a *k*-mer is queried, we can increase the counters on all bins and save the lookups in the IBF. This might reduce the size of the IBF and speed up the search time.

While not shown in this paper, the update operation on an IBF was already used in DREAM-Yara (Dadi et al., 2018) and ganon (Piro et al., 2020). Adding data is trivial since we just need to set the corresponding bits in the *x*-PIBF. For removing data from the *x*-PIBF we need to clear and rebuild the *affected* bins of the update.

### Limitations of study

The main limiting factor of our method is its susceptibility to uneven bin sizes as well as a high number of bins. The number of bins *b* directly affects the run time and memory consumption since the processed sub-bitvectors are of size *b*. While the increase in memory consumption is negligible, processing several ten thousand of bins will lead to a moderate slowdown. The size of the IBF is determined by the bin with the highest *k*-mer content. Hence, having unevenly distributed *k*-mer content across the bins will force us to increase the size of the IBF to accommodate a reasonable FP rate for the biggest bin, even though the smaller bins could achieve the same FP rate with a much lower size. However, we are actively working on a version of the IBF that will automatically adapt to inputs with uneven bin sizes. Furthermore, we will enhance the IBF by adding a hierarchical structure, i.e. a multi-level tree structure, which will dramatically reduce the number of bins needed to represent a dataset.

In conclusion, we presented a novel, versatile, fast, and memory efficient data structure for *k*-mer-based analysis of large sets of sequences using binning directories. Our implementation, Raptor, is ready for secondary memory use and its data structures can be efficiently compressed if the used bitvector is sparse. Furthermore, we showed that the concept of (w,k)-minimizers allows to effectively reduce the set of representative *k*-mers without sacrificing specificity nor sensitivity by applying our probabilistic thresholding. Raptor outperformed the state-of-the-art tools Mantis and COBS in both run time and space consumption. The use of (40,20)-minimizers was able to reduce the memory footprint of our method from 8 to 0.9 GiB for the RNA-Seq dataset introduced in (Pandey et al., 2018), which is about one order of magnitude less compared to Mantis (≈19−23 GiB). Using (w,k)-minimizers, the run time was better by factors between 12 and 144 compared to Mantis and COBS which enables completely new ways for analyzing large sequencing archives in ways that were not possible before. Raptor and binning directories are available in the SeqAn library (Reinert et al., 2017) of efficient data types and algorithms.

## STAR★Methods

### Key resources table

**Table:** 

REAGENT or RESOURCE	SOURCE	IDENTIFIER
**Deposited data**		

NIH brain, breast, and blood tissue (2652 experiments)	Pandey et al., 2018	https://doi.org/10.5281/zenodo.1186393
NCBI RefSeq Archaea and Bacteria	Various	https://doi.org/10.5281/zenodo.4647988
Clustered NCBI RefSeq (64 bins)	This paper	https://doi.org/10.5281/zenodo.4650188
Clustered NCBI RefSeq (1024 bins)	This paper	https://doi.org/10.5281/zenodo.4651078
Clustered NCBI RefSeq queries (64 and 1024 bins)	This paper	https://doi.org/10.5281/zenodo.4651379
Artificial data set (64 and 1024 bins)	This paper	https://github.com/seqan/raptor

**Software and algorithms**		

Raptor	This paper	https://github.com/seqan/raptor
Mantis	Pandey et al., 2018	https://github.com/splatlab/mantis
COBS	Bingmann et al., 2019	https://github.com/bingmann/cobs
DREAM-Yara	Dadi et al., 2018	https://github.com/seqan/dream_yara
TaxSBP	Vitor Piro	https://github.com/pirovc/taxsbp

### Resource availability

#### Lead contact

Further information and requests for resources should be directed to and will be fulfilled by the Lead Contact, Knut Reinert (knut.reinert@fu-berlin.de).

#### Materials availability

This study did not generate new materials.

#### Data and code availability

The data used for our experiments pertaining RNA-Seq is the same as used in Mantis (Pandey et al., 2018). It consists of 2652 human sequencing experiments which originate from blood, breast and brain tissue samples. A list of SRRs and URIs of these experiments is provided by the authors of Mantis via Zenodo at https://doi.org/10.5281/zenodo.1186393.

Datasets related to the biological case study using DREAM-Yara are available via Zenodo. These include: The NCBI RefSeq of archeal and bacterial genomes as of February 14th 2021 17:09 CET (https://doi.org/10.5281/zenodo.4647988), the version clustered into 64 bins (https://doi.org/10.5281/zenodo.4650188), the version clustered into 1024 bins (https://doi.org/10.5281/zenodo.4651078), and the queries used for both versions (https://doi.org/10.5281/zenodo.4651379).

All scripts and supplemental code used in this paper, including those related to the artificial dataset and DREAM-Yara, are available at https://github.com/seqan/raptor.

Pre-built DREAM-Yara indices for parameters shown in this paper are available at https://ftp.imp.fu-berlin.de/pub/raptor/.

Raptor is written in C++20 using the SeqAn Library (Reinert et al., 2017) and available at https://github.com/seqan/raptor.

### Method details

#### (w,k)-**Mminimizers**

In this work we introduce the concept of (w,k)*-minimizers* for computing representative *k*-mers. In Figure 1, we show an example for this concept. The reverse complement sequence is denoted in lower case, and we used the lexicographically smallest *k*-mer for clarity. In practice, this leads to a skewed distribution of minimizers which can be corrected by, for example, applying an XOR operation with a random value to each *k*-mer hash value before taking the numeric minimum (see (Marçais et al., 2017) for a discussion). In the Figure you see a short example of a) ungapped 4-mers in a window of size 4, which means we take the lexicographically smallest of the *k*-mer and its reverse complement as the minimizing *k*-mer. The second case b) shows the minimizing 4-mers for a window of size 8. We form the minimum of all *k*-mers and their reverse complement in this window. We denote the window span with ’-’ and place the minimizing *k*-mer at the respective window position. For the properties and the size of the data we will handle, *k* will usually be in the range of 16−32.

#### Effective text size and ratio

In general, Raptor assumes that we have a collection of strings $\left\{T_{j}\right\}$ over an alphabet Σ, with a total length $n=\underset{j}{\sum }\left|T_{j}\right|$. Raptor stores the *k*-mer content of $\left\{T_{j}\right\}$ or a *representative* transformation of it. Raptor uses (w,k)*-minimizers* for computing the set of representative *k*-mers.

To capture the repetitiveness of the $\left\{T_{j}\right\}$, we define the *effective* text length n(k) as the number of distinct, *representative k*-mers in all the $T_{j}$. In order to store the set of texts $\left\{T_{j}\right\}$, we further assume that we have divided the $T_{j}$ into *b* bins $B_{i}$ (mind that a single $T_{j}$ itself could be divided into several bins without many adaptations). For the strings in a bin $B_{i}$, we denote the set of representative *k*-mers with $B_{i}\left(k\right)$ and the effective text length with $n_{i}\left(k\right)$ as the number of representative *k*-mers of the strings in $B_{i}$, i.e. the cardinality of $B_{i}\left(k\right)$.

Dividing the strings into bins could result in a large or small intersection of their representative *k*-mer content, depending on the method. To capture this, we define the *effective text ratio* $r_{i}\left(k\right)$ as $\frac{\sum_{i}n_{i}\left(k\right)}{n\left(k\right)}$. The effective text ratio is a measure of how well we have partitioned our *k*-mer content into the bins. Ideally it is 1 and in the worst case it is *b*. We want to point out that the effective text length n(k) is a crucial measure for the problem of indexing large genomic text collections. For example, (Bradley et al., 2019) compute an index for 170 TiB of sequence data. However, this data set is quite repetitive since its effective text length n(31) is only $6\cdot 10^{10}$.

For our artificial data set, we give the effective text ratio r(k) for different *k* for both 64 and 1,024 bins in Table 8.

One can see that we need a *k*-mer size of at least 16 to achieve an effective text ratio under 2. For k≥19 the effective text ratio is near 1 which means that most *k*-mers in the bins are unique. For this reason we used k=19 in our experiments.

#### Binning directories

We define a *binning directory (BD)* for the text collection $\left\{T_{j}\right\}$ divided into *b* bins $B_{i}$ as a data structure that returns the counts of the representative *k*-mers in the query multiset I(k) for each bin $B_{i}$. In this work a binning directory uses a set membership data structure, namely the *x*-PIBF, that returns the bin membership as a (compressed) bitvector which we call the *binning bitvector*. The BD then combines the binning bitvectors into count vectors. Our Tool Raptor uses (probabilistic) thresholding to determine whether a query is in a bin or not.

Implementing a simple version is indeed not difficult. The problems lie in the fact that the effective text size n(k) can be very large, i.e. $10^{10}$ to $10^{12}$. For example, the metagenomics data set used in (Dadi et al., 2018) contains about $2\cdot 10^{10}$ different 19-mers. A naive implementation that stores all those 19-mers in a hash table containing the binning vectors for 1,024 bins would need about 40 TiB (an open addressing hash table with about $4\cdot 10^{10}$ entries, each pointing to a 1,024 bit bitvector). Hence, the challenge is implementing the BD in a more space-efficient way while maintaining a fast run time.

We approach this problem in two ways in this paper. For implementing a binning directory, we adapted the IBF data structure presented in (Dadi et al., 2018) to work well on secondary memory. We call it the *x*-partitioned IBF (*x*-PIBF). Secondly, we employ (w,k)-minimizers to reduce the number of representative *k*-mers significantly while still accurately answering the question in which bins a query can occur.

#### Answering a query with Raptor

Answering a query includes the retrieval of the binning bitvectors and the counting of *k*-mers to determine the bins to be searched. Using a *x*-PIBF, Raptor has to compute *h* hash functions, retrieve *h* sub-bitvectors and compute a bitwise AND. We can use a standard bitvector of size *n* that uses *n* bits, or the compressed bitvector of the SDSL (Gog et al., 2014) that uses approximately $m\cdot \left(2+\text{log}\frac{n}{m}\right)$ bits, where *m* is the number of bits set and *n* the length of the bitvector.

For counting, Raptor has to traverse the binning bitvector of size *b* and increment counters for each bit set to 1. To speed up this crucial step we used, for uncompressed bitvectors, the lzcount intrinsic operation which counts the number of leading zeros in a 64 bit word. This accelerated the bin counting step by a factor of almost 2 compared to the individual checking of each bit. A further speed up is possible once the AVX512 SIMD extensions are available on standard computers (already possible for Intel’s Skylake processor). These optimizations cannot be directly applied to compressed bitvectors.

Having the counts, we apply a thresholding according to the original *k*-mer counting lemma (Jokinen and Ukkonen, 1991) or according to a probabilistic model for (w,k)-minimizers.

##### Lemma 1

For a given k and number of errors e, there are $k_{p}=\left|p\right|-k+1$ many k-mers in p and an approximate occurrence of p in T has to share at least $t=\left(k_{p}-k\cdot e\right)$ k-mers.

Hence, if the count exceeds the threshold for the bin, we report the pattern to occur in this bin, otherwise not. This approach is depicted in Figure 2. However, using minimizers makes the direct application of Lemma 1 impossible for w>k. We present a solution in the next section.

#### Probabilistic thresholding

Lemma 1 works only for (k,k)-minimizers. It does not hold for general (w,k)-minimizers. The latter is apparent since the *number* and *distribution* of (w,k)-minimizers is sequence dependent and hence leads to different thresholds for each read. This problem is exemplified in Figure 3. The examples show that an error does not only *directly* invalidate the minimizers covering the error position but also *indirectly* affects minimizers *not* covering the error position, resulting in a different count of minimizers.

Taking these indirectly destroyed minimizers into consideration, there are several ad hoc ways to compute the threshold. The first is to adapt Lemma 1 such that we compute the threshold as follows: For a given *k*, *w* and number of errors *e*, there are $w_{p}=\;\left|p\right|-\;w+1$ many windows in *p* and if we take the multiplicity of the minimizers into account, an approximate occurrence of *p* in *T* has to share at least $t=\left(w_{p}-w\cdot e\right)$ minimizers, i.e. we replace *k* with *w*. However, this leads to low thresholds. The threshold in Figure 3 a) would be negative, i.e. 13−8+1−8=−2, and thus useless for filtering.

Another way to compute an individual threshold is to repeat the following two steps for each error: 1) compute the minimizer coverage of a query *p* (counting each minimizer only once) 2) One maximum coverage position is chosen and the minimizers covering this position are removed. The overall number of removed minimizers is subtracted from the number of minimizers to obtain the threshold *t*. This works better than the first approach, but is time-consuming to compute.

We can show that a simple probabilistic model yields thresholds that are on average much better than the above ad-hoc methods and removes the need to compute an individual threshold for each read. For this, we proceed as follows. We refer to the size of the query as *p*, the size of the window in which we compute minimizers is denoted by *w*, and *m* is the number of minimizers in a query. As pointed out above, an error affects several minimizers depending on its position and the character that is replaced. Each error lowers the threshold of the counting lemma. We want to derive a probabilistic model that computes this threshold for a given *p* as good as possible with only *w*, *k*, and *m* as input. We aim at having an as high as possible threshold without missing too many hits (i.e. having false negatives).

For this, we use the following definitions:

• $X_{i}$ : Random variable that is 1 if there is a minimizer starting at index *i*, ∀i∈[0,p−k+1].
• $D_{n}$: Random variable that indicates that *n* minimizers are affected by at least one error, ∀n∈[0,p−k+1].
• *τ*: Probability threshold for the number of affected minimizers.

#### Index based model for one error

In order to define $P\left(D_{n}\right)$ for one error, we need the distribution of $X_{i}$. In practice, assuming an uniform distribution of the minimizers in [0,p−k+1] yields good results:

(Equation 1) $$P\left(X_{i}\right)=\frac{\text{number of minimizer}}{\text{number of indices}}=\frac{m}{p-k+1}$$

This can now be used to compute $P\left(D_{n}\right)$. Let *j* be the position within a sequence where the error is located. Thus, it can affect at most *k* minimizers *directly*. The minimizers that are affected by the error at position *j* are the minimizers starting at indices j−i, ∀i<k. Naturally, these minimizers are the only ones that include the position *j*. Hence, there are at most *k* minimizers that include the position *j* and thereby there are at most *k* minimizers *directly* modified by the error. This can be expressed via a binomial distribution and yields:

(Equation 2) $$\begin{array}{l} \forall n\;\in \left[0,k\right]:\\ P\left(D_{n}\right)=\left(\begin{array}{l} k\\ n \end{array}\right)P{\left(X_{i}\right)}^{n}{\left(1-P\left(X_{i}\right)\right)}^{k-n}=\left(\begin{array}{l} k\\ n \end{array}\right){\left(\frac{m}{p-k+1}\right)}^{n}{\left(1-\frac{m}{p-k+1}\right)}^{k-n} \end{array}$$

$P\left(D_{n}\right)$ can now be used to compute the new filtering threshold. Let *d* be the number of (w,k)-minimizers affected such that

(Equation 3) $$\overset{d}{\underset{i=0}{\sum }}P\left(D_{i}\right)>\;\tau \text{ and }\overset{d-1}{\underset{i=0}{\sum }}P\left(D_{i}\right)<\;\tau$$

Then the following lemma holds:

##### Lemma 2

For a given p, w, and k and one error, at most d many k-mers are affected with a probability of τ. Thus, an approximate occurrence of the query in T has to share at least t=m−d k-mers with probability at least 1−τ.

This method enables us to use *τ* to control the false positive and false negative rates of our filter. The higher we choose *τ*, the more minimizers are affected, i.e. the threshold *t* decreases and the false positive rate increases. The lower we choose *τ*, the fewer minimizers are affected, i.e. the threshold increases *t* and the false negative rate decreases.

Furthermore, this model only depends on *m* which is known for each read. In order to obtain the threshold, we simply have to look it up in a precomputed table for the specific parameters or compute the table once if it has not been computed yet.

#### Extension for indirect errors

The previous model can be extended to account for errors affecting minimizers *indirectly*. The example in Figure 3 shows that an error can affect a minimizer even though the error does not overlap with the minimizer.

We define the random variable $C_{n}$ that is 1 if the error affects *n k*-mers indirectly. Thus, if we assume for simplification that the events of directly and indirectly affecting a minimizer are independent, $P\left(D_{n}\right)$ becomes:

(Equation 4) $$\begin{array}{ll} \forall n\;\in \left[0,w\right]: & \\ & P\left(D_{n}\right)=\overset{w-k}{\underset{i=1}{\sum }}P^{\text{′}}\left(D_{n-i}\right)P\left(C_{i}\right)\\ \text{with} & \\ & P^{\text{′}}\left(D_{n}\right)=\left\{ \begin{array}{ll} P^{\text{′}}\left(D_{n}\right) & \text{ if }n\;<=k\\ 0 & \text{else} \end{array}\right. \end{array}$$

where $P^{\text{′}}\left(D_{n}\right)$ is the distribution of the previous model.

The main challenge with this extension is the computation of $P\left(C_{n}\right)$. We tried to find a tractable formulation of $C_{n}$, but did not succeed and leave this as an open problem. This is why we estimate $P\left(C_{n}\right)$ in this work experimentally by sampling. We generate a query with and without an error and then check the number of minimizers that are indirectly modified by the error. This sampling method is flexible for different parameters and can be easily applied for the relevant range of parameters or be quickly computed on the fly. In our experiments, a sampling of 10,000 cases lead to convergence of $P\left(C_{n}\right)$. The thresholds are computed for τ=0.99.

#### Extension for multiple errors

We can further extend our model to consider multiple errors. For example, assume that there are 2 errors, $e_{1}$ and $e_{2}$, that affect $d_{1}$ and $d_{2}$ many *k*-mers, respectively. Thus, the two errors combined affect at most $d=d_{1}+d_{2}$ many *k*-mers. Hence, affecting at most *d* many *k*-mers can be achieved by all different combinations of $\left(d_{1},d_{2}\right)$ such that $d\;\geq d_{1}+d_{2}$ and $0\;\leq d_{1}\leq w$, $0\;\leq d_{2}\leq w$.

Generalizing this for *e* errors affecting $d_{1},\ldots ,d_{e}$ minimizers leads to the following distribution:

(Equation 5) $$\begin{array}{ll} \forall n\;\in \left[0,w\cdot e\right]: & \\ P\left(D_{n}\right) & =\overset{d_{1}+d_{2}+\ldots +d_{e}=n}{\underset{0\leq d_{1}\leq w,\ldots ,0\leq d_{e}\leq w}{\sum }}\overset{e}{\underset{i=1}{\prod }}P^{\text{′}}\left(D_{d_{i}}\right) \end{array}$$

with $P^{\text{′}}\left(D_{n}\right)$ being the distribution of the previous model allowing for indirect errors.

We also developed more involved models to account for two errors being in close vicinity (not shown), however the rounded thresholds very seldomly change. Hence, we omit them.

In summary, the threshold for the counting lemma can be looked up in a table given the number of errors, the number of minimizers of a query, and the parameters (w,k) and hence the filtering speeds up considerably. The method models the occurrences of (w,k)-minimizers within the windows and how they affect each other (see Figure 3 for an example).

#### *x*-partitioned IBF

Finally, we propose our last contribution, the use of an *x*-partitioned interleaved Bloom filter (*x*-PIBF) in binning directories, which are an extension of the IBF proposed in (Dadi et al., 2018). An IBF for *b* bins combines *b* standard Bloom filters (Bloom, 1970). A Bloom filter is simply a bitvector of size *n* and a set of *h* hash functions that map a value, in our case a representative *k*-mer, to one of the bit positions. A value is present in the Bloom filter if all *h* positions return a 1. Note that a Bloom filter can give a false positive answer. However, if the Bloom filter size is large enough, the probability of a false positive answer is low. A Bloom filter of size *n* bits with *h* different hash functions and *m* elements inserted has a probability of giving a false positive answer of approximately

$$p_{fp}={\left(1-{\left(1-\frac{1}{n}\right)}^{h\cdot m}\right)}^{h}.$$

For this reason, we have to allocate sufficient space such that $p_{fp}$ does not become too large. Still, the problem of using a simple Bloom filter is that it does not point us to the binning bitvectors. To alleviate the problem, the IBF uses *b* Bloom filters (one for each bin) with identical hash functions and then interleaves their bitvectors. Putting it differently, this means that it replaces each bit in the Bloom filter by a (sub)-bitvector of size *b*, where the *i*-th bit ”belongs” to the Bloom filter for bin $B_{i}$. The resulting IBF has a size of b⋅n. When inserting a *k*-mer from bin $B_{i}$ into the IBF, it computes all *h* hash functions which point to the position of the block where the sub-bitvectors are and then sets the *i*-th bit from the respective beginnings. Hence, the IBF effectively interleaves *b* Bloom filters in a way that allows us to easily retrieve the binning bitvectors for the *h* hash functions. When querying in which bins a *k*-mer can be found, we retrieve the *h* sub-bitvectors and apply a logical AND to them which results in the required binning bitvector indicating the membership of the *k*-mer in the bins. The procedure is depicted in Figure 4.

Finally, the binning bitvectors are summed up to obtain the count vectors. For this, we allocate *t* many counters, each with *b* entries, where *t* is the number of threads used. These counters are reused as we process the reads in parallel.

If the set of stored *k*-mers is very large or if we want to achieve a very low FPR of the IBF, it might be too big to keep in the main memory. For those cases we implemented the *x*-partitioned IBF (*x*-PIBF) where we partition the set of stored *k*-mers into *x* parts as follows:

For a *x*-PIBF of size *s* bits, we create *x* parts, each with [s/x] bits. Then we partition the *k*-mers based on the first *q* characters with $q\;\geq {\text{log}}_{\sigma }\;\left(x\right)$, where *σ* is the alphabet size. We count the *q*-mer frequencies of all *k*-mers and assign them as evenly as possible to the *x* parts (see below table for an example). The counting step can be omitted, in which case we assume a uniform prefix distribution.

Finally, we have to adapt our hash functions such that all *h* hash values for a *k*-mer lie in the same part of the *x*-PIBF. This can easily be done by storing offsets in a $q^{\sigma }$ large table and adding those to the hash values for an IBF of size [s/x].

If we query a set of *k*-mers, we load the first part of the *x*-PIBF into memory, stream over all *k*-mers counting the relevant ones for this part and ignoring the others. Then we repeat this for all other parts after loading them. In the Results section we report on the time/memory trade-off.

#### Compressing bitvectors

Binning directories use a large bitvector containing all the binning bitvectors for all representative *k*-mers. In this work we also allow the use of a compressed bitvector implementation from the SDSL (Gog et al., 2014). While a standard bitvector of size *n* uses *n* bits, the compressed bitvector of the SDSL uses approximately $m\cdot \left(2+\text{log}\frac{n}{m}\right)$ bits, where *m* is the number of bits set, and *n* the length of the bitvector. Note that while this can reduce the space consumption for sparse bitvectors, it increases the access time which we will discuss in the experiments.

To construct a compressed bitvector, we first have to create the *entire* uncompressed bitvector and then compress it. This means that both the uncompressed and compressed bitvectors have to be in main memory at some point during construction which increases the memory footprint during construction while reducing the memory requirements when using the bitvector. A main property of the compressed bitvector is that it is immutable. If we want to change a bit after the vector is constructed, we need to change the bit in the uncompressed bitvector and reconstruct the compressed bitvector. Since decompression for the compressed bitvector is not supported by the SDSL, we also need to store the uncompressed bitvector on disk to enable future updates of the IBF. Nevertheless, we need to have the whole bitvector initially in memory which might pose a problem. This problem can be solved elegantly using the partitioning of the IBF as proposed before.

### Quantification and statistical analysis

For the evaluation of our method, we use the notions of *false negatives* (FN) and *false positives* (FP). We examine reads originating from a known bin *i*. A false positive is a read that originated from bin *i* but was assigned to any bin j≠i. If a read was assigned to multiple bins, it is likewise classified as false positive, as at least one of the reported bins must not be bin *i*. A false negative read is a read originating from bin *i* that was not assigned to bin *i*, independent of how many bins it was assigned to.
